# Phylogenetic and Molecular Variability Studies Reveal a New Genetic Clade of *Citrus leprosis virus C*

**DOI:** 10.3390/v8060153

**Published:** 2016-06-06

**Authors:** Pedro Luis Ramos-González, Camila Chabi-Jesus, Orlene Guerra-Peraza, Michèle Claire Breton, Gabriella Dias Arena, Maria Andreia Nunes, Elliot Watanabe Kitajima, Marcos Antonio Machado, Juliana Freitas-Astúa

**Affiliations:** 1 Laboratório de Biotecnologia, Centro de Citricultura Sylvio Moreira, Instituto Agronômico de Campinas, Cordeirópolis, São Paulo 13490-970, Brazil; plrg1970@gmail.com (P.L.R.-G.); millachabi@yahoo.com.br (C.C.-J.); mbreton@uol.com.br (M.C.B.); gaby.arena@gmail.com (G.D.A.); mandreian@yahoo.com (M.A.N.); marcos@centrodecitricultura.br (M.A.M.); 2 Departamento de Bioquímica Fitopatológica, Instituto Biológico, São Paulo 04014-002, Brazil; onelio@protonmail.com; 3 Departamento de Microbiologia Agrícola, Escola Superior de Agricultura Luiz de Queiroz, Universidade de São Paulo, Piracicaba, São Paulo 13418-900, Brazil; 4 Instituto de Biologia, Universidade de Campinas, Campinas, São Paulo 13083-970, Brazil; 5 Departamento de Fitopatologia e Nematologia, Escola Superior de Agricultura Luiz de Queiroz, Universidade de São Paulo, Piracicaba, São Paulo 13418-900, Brazil; ewkitaji@usp.br; 6 Embrapa Cassava and Fruits, Cruz das Almas, Bahia 44380-000, Brazil

**Keywords:** cilevirus, citrus leprosis, *Brevipalpus*-transmitted viruses

## Abstract

*Citrus leprosis virus C* (CiLV-C) causes a severe disease affecting citrus orchards in the Western hemisphere. This study reveals the molecular variability of the virus by analyzing four genomic regions (*p29*, *p15*, *MP* and RNA2-intergenic region) distributed over its two RNAs. Nucleotide diversity (π) values were relatively low but statistically different over the analyzed genes and subpopulations, indicating their distinct evolutionary history. Values of π*_p_*_29_ and π*_MP_* were higher than those of π*_p_*_15_ and π_RNA2–IR,_ whereas π*_MP_* was increased due to novel discovered isolates phylogenetically clustered in a divergent clade that we called SJP. Isolate BR_SP_SJP_01 RNA1 and RNA2 sequences, clade SJP, showed an identity of 85.6% and 88.4%, respectively, with those corresponding to CiLV-C, the type member of the genus *Cilevirus*, and its RNA2 5′-proximal region was revealed as a minor donor in a putative inter-clade recombination event. In addition to citrus, BR_SP_SJP_01 naturally infects the weed *Commelina benghalensis* and is efficiently transmitted by *Brevipalpus yothersi* mites. Our data demonstrated that negative selection was the major force operating in the evaluated viral coding regions and defined amino acids putatively relevant for the biological function of cilevirus proteins. This work provides molecular tools and sets up a framework for further epidemiological studies.

## 1. Introduction

Citrus leprosis is considered a re-emerging and serious viral disease threatening citrus production [1]. The infection decreases plant lifespan and affects fruit quality, reducing yields and increasing production costs of citrus orchards. Endemic in the Americas, leprosis was first observed in Florida, USA, São Paulo State, Brazil, and restricted areas of Argentina and Paraguay at the beginning of the 20th century [2,3,4,5]. After the 1960s, the disease disappeared from Florida [6], but in the Southern region of South America it became prevalent occurring in most of the Brazilian citrus growing areas [7,8]. More recently, Bolivia, Colombia, Venezuela, all of the Central American countries and Mexico have been incorporated to the citrus leprosis distribution map [9,10,11,12,13,14,15,16,17,18,19]. Outside of mainland America, symptoms associated with citrus leprosis have been confirmed only in Hawaii, USA [20,21].

**Figure 1:**
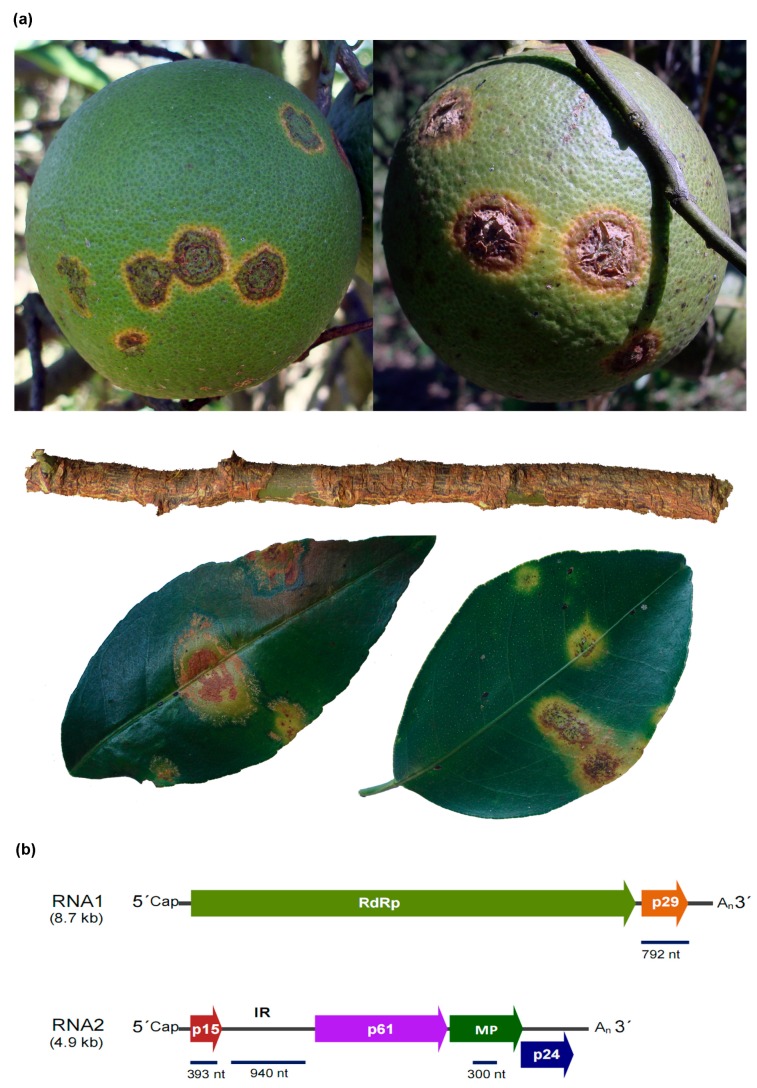
(**a**) Citrus leprosis symptoms in sweet orange (*Citrus sinensis*) fruits, twig and leaves. (**b**) Genomic organization of *Citrus leprosis virus C* (CiLV-C). Blue solid bars indicate regions used in the variability study. *RdRp*: RNA-dependent RNA polymerase; *p29*: putative coat protein; *MP*: putative 30K superfamily movement protein; *p15*, *p61* and *p24*: proteins with unknown functions. IR: intergenic region. A_n_: poly(A) tail.

Viruses causing citrus leprosis show bacilliform particles and are persistently transmitted by false spider mites of the genus *Brevipalpus* (Acari: *Tenuipalpidae*) [22,23,24,25]. They induce localized necrotic or chlorotic lesions around the mite-feeding sites and viral systemic movement has not been reported in any of their known natural or experimental hosts (Figure 1a) [26,27]. Citrus leprosis associated viruses display heterogeneous genomic features so they are taxonomically classified in three distinct genera: *Cilevirus*, *Higrevirus* and *Dichorhavirus* [20,28,29,30]. During replication these viruses induce either nuclear or cytoplasmic cellular malformations, distinguishing two forms of the disease: the nuclear and cytoplasmic types, respectively. By far these two features are the most extensively used to differentiate the citrus leprosis causal agents [22,24].

Occurrence of leprosis of the nuclear type in Panama and in some regions of Brazil was reported, although the molecular identity of the associated viruses was not identified [22]. In Mexico and Colombia, this type of the disease was reported as caused by two closely related citrus strains of the species *Orchid fleck virus* (OFV) (synonym citrus leprosis virus nuclear type (CiLV-N) and citrus necrotic spot virus (CiNSV)), which is considered the type member of the genus *Dichorhavirus* (bipartite, negative-sense (−) single-stranded (ss) RNA) [12,13,30,31]. In spite of that, the typical and prevalent leprosis in citrus orchards from Mexico to Argentina is of the cytoplasmic type caused by isolates of *Citrus leprosis virus C* (CiLV-C, type species of the genus *Cilevirus*, bipartite positive-sense (+) ssRNA genome) [22,29]. In some areas of Colombia, cytoplasmic leprosis is also caused by isolates of *Citrus leprosis virus C 2* (CiLV-C2, a proposed member of the cilevirus genus). A related isolate of CiLV-C2, found infecting ornamental hibiscus (*Hibiscus* sp.) in Hawaii (CiLV-C2 Hw), shows a global amino acid identity of 92% with CiLV-C2 [21]. In Hawaii, leprosis-like symptoms in *Citrus volkameriana* trees are associated to the presence of *Hibiscus green spot virus 2* (HGSV-2) [20], the type and single member of the genus *Higrevirus* (tripartite (+) ssRNA genome).

Each genomic component of CiLV-C contains a cap structure at the 5′ region and a poly(A) tail at the 3′-terminal. RNA1 (8.7 kb) encodes for two open reading frames (ORFs) identified as the RNA-dependent RNA polymerase and the putative coat protein (*p29*), while RNA2 (4.9 kb) encodes for four ORFs recognized as *p15*, *p61*, putative movement protein (*MP*) and *p24* (Figure 1b) [32]. MP shares a conserved domain with the 30K superfamily of viral plant movement proteins [32], and P24 and P61 proteins show a distant homology with putative structural proteins encoded by blunerviruses and negeviruses [33]. CiLV-C RNA1 acts as template for generation of the sub-genomic RNA1 (sgRNA1) of 0.7 kb specific for *p29* gene translation and RNA2 generates another three 3′-coterminal sgRNAs within 3 kb (sgRNA2), 1.5 kb (sgRNA3) and 0.6 kb (sgRNA4), probably involved in the expression of *p61*, *mp* and *p24* genes, respectively [34]. RNA2 harbors a large intergenic region (IR) of circa 1130 nt in length, located between the *p15* and *p61* genes. Overall, CiLV-C2 shows a similar genome organization to CiLV-C and globally their genomes share 55% nucleotide identity. The lowest values of nucleotide identity among these viruses correspond to the stretch covering the *p15* and IR in the RNA2 molecule [21].

Plant to plant transmission of viruses causing the cytoplasmic type of leprosis is known to be mainly mediated by the mite species *Brevipalpus yothersi* Baker (synonym *B. phoenicis* Geijskes citrus type) [1,25]. *Brevipalpus* populations are mostly composed of females which reproduce by thelytokous parthenogenesis [35]. *B. yothersi* is worldwide distributed, polyphagous, and after a short period of virus acquisition during any of the four active phases of its life span, persistently transmits CiLV-C [36,37]. Evidence of transovarial transmission of the virus in mites has not been obtained, whereas viral multiplication in the vector has been suggested [1].

Absence of proofreading activity in the RNA-dependent RNA polymerases leads to high mutation rates and potential generation of genetic diversity in RNA viruses [38,39]. Additionally, variability could be also introduced by recombination and reassortment among the segments of split genomes [40,41,42,43,44]. However, continuous constraints on the population by bottlenecks both within hosts and when the virus is plant-to-plant transmitted may account for a genetic diversity lower than expected, as verified for many plant virus species [45]. At the same time, changes in biotic or abiotic factors (e.g., interaction with new hosts or vectors) shape the virus population allowing the emergency of low frequency haplotypes. Those better adapted to the novel imposed selection pressures could be the potential origin of novel epidemics [46,47].

Leprosis is considered the main viral disease affecting citrus orchards in Brazil, the leading sweet orange producer in the world [48]. Considering that extensive understanding about pathogens may positively impact disease management strategies [47], we examined the population of leprosis associated viruses in Brazil. As a result, we identified the presence of CiLV-C as the only virus causing citrus leprosis in all analyzed samples, and detected the existence of a second clade within the screened population. Complete genomic sequence of one member of the new clade was obtained and characterized. The study identified codons under selection in the *p29*, *p15* and *MP* genes, which might be relevant during the replication cycle of cileviruses. It also allowed us to assess the genetic variability of CiLV-C and provided an insight into the evolutionary history of cileviruses. Moreover, we described primer pairs to differentially detect the presence of CiLV-C isolates belonging to the two phylogenetic clades identified in this work giving support for further epidemiological studies.

## 2. Materials and Methods

### 2.1. Leprosis Surveys, Virus Detection and Isolates

A total of 48 samples collected during 2012–2015 were analysed in this study (Table 1). Among them, 45 samples corresponded to citrus (*Citrus sinensis* L. Osb.) leaves exhibiting necrotic or chlorotic symptoms were collected in commercial orchards or small private yards in all geographic regions throughout Brazil. A citrus sample from Argentina and two tropical spiderwort (*Commelina benghalensis*) plants exhibiting symptoms of CiLV-C infection [49] were also included. Symptomatic leaf tissues were cut, ground in liquid nitrogen and kept at −80 °C until further processing.

All samples were assayed for detection of CiLV-C, CiLV-C2 and the citrus strain of OFV (synonym CiLV-N). Total RNA was obtained from approximately 100 mg of leaves using TRIZOL^®^ Reagent and following the manufacturer’s recommendations (Life Technologies, Foster City, CA, USA). cDNA (500 ng of total RNA) were generated using RevertAid H Minus First Strand cDNA Synthesis Kit as described by the manufacturer (Thermo Scientific, Madison, WI, USA), using random hexamer primers. Three to five µL of the cDNA solution were taken for PCR using specific primers for the detection of CiLV-C, CiLV-C2 and CiLV-N [14,31,51]. PCR products were separated on 1.0% agarose gels in 1X Tris-acetate-Ethylenediaminetetraacetic acid (TAE) and visualized with ethidium bromide (0.1 μg/mL).

### 2.2. Sequencing of the RNA2-Intergenic Region and p29, p15 and Partial MP Genes of CiLV-C Isolates

To assess the molecular variability of CiLV-C, fragments of 864 nt spanning the ORF *p29* in RNA1, were amplified using the primer pair (5′-ATGAGTATCGTAACTTTCACTTTGAC-3′/5′-ACCAGAGATTAGCGATTCAAAA-3′). In RNA2, amplicons of 667 nt containing the ORF *p15* were amplified with primers (5′-TGTTCTAGGCTAATAACTCTCAA-3′/5′-CTGAAACAGCTCATGAAACA-3′), and for the IR fragments of *ca.* 980 nt the following primers (5′-ACTTGTGTTTGTCATTTGCC-3′/5′-GCTTGATTTGTTGTAGGCTC-3′) were used. Primers were designed using Primer3 [52] based on the CiLV-C genome sequence (GenBank accession numbers DQ352194 and DQ352195) [32] (Figure 1b). Amplification was carried out in a 20 µL reaction containing 2 mM of corresponding primers, 1 U of GoTaq polymerase (Promega, Madison, WI, USA), 10 mM of deoxynucleotides (dNTP) mix and 25 mM of MgCl_2_. The thermal cycling was as follows: one cycle of 95 °C for 5 min and 35 cycles of 95 °C for 30 s, 54 °C for 30 s, 72 °C for 1 min. An additional final extension for 10 min at 72 °C was performed. Amplicons were purified using Promega Wizard SV and PCR clean-up system, ligated in the pGEM-T-easy vector (Promega) and transformed into chemically competent *Escherichia coli* DH5α. Recombinant plasmids were purified from bacteria cells using Wizard^®^ Plus SV Minipreps DNA Purification System (Promega) and *Eco*RI digested to confirm the cloning of the expected amplicons. Three to six clones per each isolate and viral genome region were selected. Amplicons were sequenced using both the primers that generated them and the pUC/M13 forward and reverse primers by the Sanger method with the BigDye Terminator 3.1 kit (Perkin Elmer, Waltham , MA, USA) in an automated sequencer ABI Prism 3730 (Applied Biosystems, Foster City, CA, USA). No polymorphic sequences were detected in amplicons derived from each sample, indicating absence of mixed infections.

### 2.3. Phylogenetic Analysis and Estimation of Population Genetic Parameters

Amplicon sequences were trimmed removing primer information. Multiple sequence alignments were performed using MUSCLE algorithm [53] implemented in the Molecular Evolutionary Genetic Analysis (MEGA) software version 6.0 for Windows [54]. Best fit nucleotide substitution models for each dataset were also estimated using MEGA6. Models with lowest Bayesian information criterion (BIC) score for *p29*, *p15*, *MP*, IR and the segment of the RNA2 encompassing the concatenated sequences of *p15*, IR and *MP* were Kimura-2 parameter + gamma distribution (K2 + G), Tamura 3-parameter (T92), K2, T92 + G, and Tamura Nei (TN93), respectively. Models were used for the estimation of nucleotide distances and phylogenetic relationships. Mean values of the genetic distances calculated within and between defined clades were compared using a Student´s *t*-test. Phylogenetic trees were inferred using both the neighbor-joining (NJ) and maximum likelihood methods using sequences generated in this work or available in the GenBank (Table 1). The robustness of the inferred evolutionary relationships was assessed by 1000 bootstrapped replications. Nucleotide sequences from CiLV-C2 isolates Colombia and Hawaii were used as the out-groups for these analyses. The generated trees were edited using FigTree version 1.4.2 [55]. Number of haplotypes (h), nucleotide and haplotype diversities(π and Hd, respectively) and the confidence intervals for π values (based on coalescent simulations, 10,000 replicates) were estimated using DnaSP software (version 5.10.01) [56].

### 2.4. Estimation of Natural Selection Pressure

Selection pressure on coding regions of CiLV-C (full length *p29* and *p15* genes and partial *MP* gene) was estimated calculating the ratio (ω) of non-synonymous (dN, non-conservative mutations)/synonymous (dS, silent mutations) nucleotide substitutions. dN and dS were estimated in MEGA6 [54] using the bootstrap method (1000 replicates) and the Kumar model. Sites under selection were detected with SLAC (single likelihood ancestor counting), FEL (fixed effects likelihood) and REL (random effects likelihood) methods implemented in the HyPhy package [57,58]. Each method was run using the default cut-off values of probabilities and codons were considered under selection when indicated by at least two methods. The best-fit substitution model for each set of sequences was selected by the software and each haplotype was represented only once in the analyses. Before the analyses, alignments were assessed using a genetic algorithm for recombination detection (GARD) [59] also implemented in the HyPhy package.

### 2.5. Recombination Analyses

Putative recombination events amongst CiLV-C isolates were evaluated using different methods, namely recombination detection program (RDP), statistical tests for detecting gene conversion (GENECONV), MaxiChi, Chimera, SiScan, and 3Seq, implemented in the RDP Beta 4.66 [60]. Alignments of nucleotide sequences (*p29* or those as result of concatenation of *p15*-IR-*MP*) produced in MEGA6 [54] were run in RDP4 using the default settings (0.05 *p*-value cut-off, standard Bonferroni correction, and the option ‘Reference sequence selection’ set as ‘internal references only’). Concatenated sequences derived from CiLV-C2 Colombia RNA2 were also included in this analysis to assess putative inter-species recombination. To omit unreliable signals, only those recombinant events supported by four or more methods were taken into account.

### 2.6. Secondary Structure Prediction of P29 Proteins of Cileviruses

Predicted secondary structures of deduced amino acid sequences of the putative coat proteins (P29) from definitive and tentative members of the genus *Cilevirus i.e.*, CiLV-C (GenBank accession No. ABC75822), CiLV-C isolate BR_SP_SJP_01 (GenBank accession No. AKJ79133), CiLV-C2 isolate Colombia (GenBank accession No. ABA42876), and CiLV-C2 isolate Hawaii (GenBank accession No. AGM16552) were obtained using PROMALS (PROfile Multiple Alignment with predicted Local Structure) [61]. The multiple alignment also included the P29 deduced amino acids sequences from several CiLV-C isolates identified in this work and was done using T-coffee [62]. Secondary structure information and T-coffee alignment were edited using ESPript [63]. PROMALS and T-coffee parameters were set as default.

### 2.7. RT-PCR Test for Differential Detection of CiLV-C Clade-Specific Isolates

In order to differentially detect viruses of the clade SJP from those CiLV-C predominant isolates, two specific primer pairs for the *p29* genes of each kind of isolate were designed. Forward primers spanned a six nucleotide insertion observed in the *p29* of the CiLV-C isolates from the clade SJP. Target regions for reverse primers were conveniently selected in order to generate isolate-specific size amplicons. Primer sequences were as follow: p29CRD-F: ^7938^CAGAAGGCCGAGGTTGTAAAG^7958^, p29CRD-R: ^8267^GTAGTGATCACTGAACTCGAATACC^8244^, p29SJP-F: ^7931^GTAAACAAAAGGTCGAGGTTGTCC^7954^ and p29SJP-R: ^8386^TCTGTTGTCTAGCAGCAAGTAATG^8363^. The abbreviations SJP or CRD in the name of primers indicate their specific targets, members of the clade SJP or the prevalent CiLV-C isolates, respectively. Validation of the test was performed with samples including isolates belonging to the two lineages and infecting different host plants (Table 1). RT-PCR assays were carried out as aforementioned. PCR products were purified and sequenced to confirm their identities.

### 2.8. Characterization of the Isolate BR_SP_SJP_01

For the complete genome sequencing of the isolate BR_SP_SJP_01, total RNA was extracted from approx. 500 mg of symptomatic sweet orange leaves collected from a tree in São José do Rio Preto, SP, Brazil; or *Arabidopsis thaliana* plants infested with mites reared onto symptomatic sweet orange fruits collected in the same tree. Samples were ground in presence of liquid nitrogen and total RNA extracts were obtained using TRIZOL^®^ Reagent according to the manufacturer’s recommendation (Life Technologies). In the last washing step, ethanol 75% solution was replaced by ethanol 100%. RNA quantification and A_260_/A_280_ ratios were estimated using the NanoDrop ND-8000 micro-spectrophotometer (Thermo Scientific, Waltham, MA, USA). Integrity of samples was verified by a Bioanalyser 2100 device (Agilent Technologies, Santa Clara, CA, USA). Samples were shipped to FASTERIS (Plan-les-Ouates, Switzerland), where small RNAs were separated and processed for sequencing using the high throughput Illumina GA IIX platform. High throughput sequencing of the small RNA fraction libraries of 20–25 nt from sweet orange and Arabidopsis plants generated 4,693,816 and 2,264,321 reads; respectively (Table S1). Reads ranging from 20 to 25 nt from infected sweet orange and Arabidopsis were independently assembled in contigs using the Velvet Assembler [64]. After the screening in the *A. thaliana* [65] and citrus (*C. sinesis*_v1.0) [66] genome sequence databases, contigs containing plant-derived information were excluded from the analysis and the rest were assembled using CLC genomics Workbench 6.05 [67] and the CiLV-C genome (GenBank accession numbers DQ352194 and DQ352195) as reference [32]. All sequences were assembled together and a set of 34 primer pairs (Table S2) were designed from the obtained scaffold using Primer3 [52]. cDNA synthetized from the diseased sweet orange RNA extract was used to generate overlapping amplicons of approx. 600 nt in length in order to close the sequencing gaps and confirm the next generation sequencing data. Amplicons were cloned and sequenced as formerly described. After final assembly, the full genome sequence of CiLV-C isolate BR_SP_SJP_01 was deposited in the GenBank database (accession numbers KP336746 and KP336747).

Using the genome of the isolate BR_SP_SJP_01 as reference, it was determined that 51.3% and 74.2% of 20–25 nt reads in the sweet orange and Arabidopsis libraries, respectively, mapped to the viral genome. Reads from sweet orange covered 98% and 86% of the RNA1 and RNA2, respectively; and those obtained from Arabidopsis provided 94% of the RNA1 and 98% of the RNA2 (Table S1). In both libraries, the most abundant size class of viral derived small RNAs had 21 nt in length, followed by a second peak of 24 nt.

Transmission of the isolate BR_SP_SJP_01 was achieved using a *B. yothersi* isoline population obtained from a single female collected in Bahia State, Brazil. Unripe sweet orange fruits of *cv.* Pera were used as substrate for maintenance of non-viruliferous mite populations. Fruits were cleaned, dried, partially submerged in liquid paraffin to prevent desiccation, and surrounded by the pest adhesive barrier Biostop gum (Biocontrole, Indaiatuba, SP, Brazil). For CiLV-C isolate BR_SP_SJP_01 transmission, symptomatic orange fruits collected in São José do Rio Preto, SP were treated as described for the healthy ones, allowing the exposure of the symptomatic areas as feeding arenas. Healthy and diseased fruits were placed side by side to allow mite migration and further virus acquisition for a minimal period of three days. For virus inoculations in Arabidopsis and sweet orange plants, five adult viruliferous mites were transferred, with a small brush under a stereoscopic microscope, to each of three leaves per plant (rosette leaves in the case of Arabidopsis). Fifteen Arabidopsis and ten sweet orange plants were assayed in each of the three independent transmission experiments. As control, a plant of each species was inoculated with the same amount of mites from the non-viruliferous colony. After symptoms appeared, viral presence was confirmed by RT-PCR using primers specific for the detection of ORF *MP* [51] and clade SJP-ORF *p29* (formerly described in Section 2.7). Arabidopsis Col-0 plants used in this experiment were obtained from seeds, which were planted in pots and kept at 4 °C in the dark. After four days, plants were allowed to grow at 22 ± 2 °C with a 12 h light cycle in an Adaptis-1000 environmentally controlled growth chamber (CONVIRON, Winnipeg, MB, Canada). Seedlings of sweet orange cv. Pera obtained from seeds were kept under greenhouse conditions.

## 3. Results

### 3.1. CiLV-C Is the Prevalent Causal Agent of Citrus Leprosis in Brazil

The presence of citrus leprosis associated viruses was evaluated in a discrete set of 46 symptomatic sweet oranges trees of which 56% were from São Paulo State, the main sweet orange producing region in the world [48]. None of the samples yielded PCR products for either CiLV-N or CiLV-C2, but all of them produced the expected 339 bp fragments when amplified using the specific primers to detect CiLV-C [51] (Figure S1). For further phylogenetic and population studies of CiLV-C, in addition to the *MP* gene fragment used for viral detection, another three genomic regions (complete *p29* gene in the RNA1, and complete *p15* and IR in the RNA2, Figure 1b) were amplified from 33 samples covering 22.3% of the viral genome. All recovered sequences and others retrieved from the GenBank database were compiled and, in total, 48, 47, 41 and 38 sequences were used in the *p29*, *MP*, *p15* and IR analyses, respectively (Table 1).

### 3.2. Phylogenetic Analysis of the p29 and MP Genes Indicate a Second Clade within the CiLV-C Population

**Figure 2:**
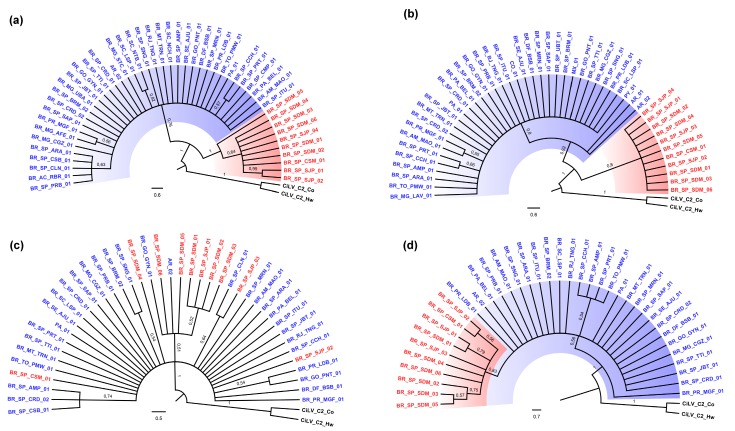

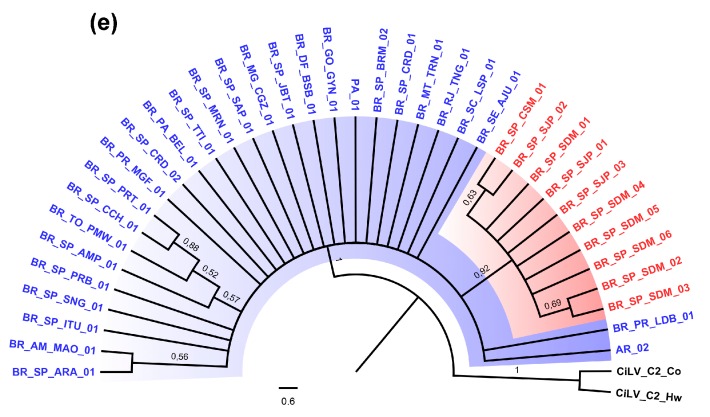
Midpoint-rooted neighbor joining phylogenetic trees of CiLV-C isolates based on four genomic regions. Isolates of the clade CRD and SJP were highlighted in blue and red, respectively. (**a**) *p29* complete sequence (792 nt); (**b**) *MP* partial sequence (300 nt); (**c**) *p15* complete sequence (393 nt); (**d**) IR (940 nt); (**e**) Fragment encompassing concatenated sequences of the RNA2 (*p15* + IR + *MP*, 1633 nt). Sequences from CiLV-C2 isolate Colombia (CiLV-C2_Co, GenBank accessions JX000024 and JX000025) and CiLV-C2 isolate Hawaii (CiLV-C2_Hw, GenBank accessions KC626783 and KC626784) were incorporated as outgroup. Bootstrap support values (1000 iterations) of main branches are indicated. Branches with less than 50% bootstrap support were condensed.

Phylogenetic trees inferred from the complete nucleotide sequence of *p29* and the partial sequence of the *MP* (300 nt) using either neighbor joining and maximum likelihood methods showed the occurrence of two divergent clades within the CiLV-C population (Figure 2a,b). In these trees, most of the isolates grouped together with those with known full-length genomes *i.e.*, CiLV-C isolates PA01 and BR_SP_JBT_01 [32,34] and particularly with the isolate BR_SP_CRD_01, the type member of the cilevirus genus and up to now recognized as the only genotype of the species [8,22]. This group was called CRD, abbreviation used to refer to the Cordeirópolis city in the São Paulo State, Brazil, place of collection of the isolate BR_SP_CRD_01 [32]. This abbreviation will be used to specify this viral lineage from now on. The smaller branch of each tree grouped isolates collected in São José do Rio Preto (SJP), Cosmorama (CSM), and Sud Mennucci (SDM), three localities situated *ca.* 100–300 km distant from each other in the central–Northern part of São Paulo State. This new clade was named SJP denoting the homonymous city, place of the collection of four of the included isolates.

In the *MP* gene-based tree most of the Brazilian isolates and those from Mexico (MX_01), Colombia (CO_01), Panama (PA_01), Paraguay (PY_01) and Argentina (AR_01 and AR_02) were grouped in the same branch. The two isolates from tropical spiderwort plants, identified as BR_SP_BRM_01 and BR_SP_SJP_04, were separately distributed in the two predefined branches (Figure 2b).

### 3.3. p15 and IR-Based Trees Suggest Recombination

Topologies of the generated trees using *p15* and IR genomic regions were different from those observed using *p29* and *MP*. *p15* tree failed to separate the isolates in the two previously detected branches, probably indicating the low diversity of this gene (Figure 2c). Some members of the formerly defined clades were clustered together (*i.e.*, isolate BR_SP_SJP_03 of the clade SJP with the isolate BR_SP_CLN_01 of clade CRD, respectively), although in a relatively low bootstrap supported branch (64% of trees). In the IR tree, predefined clades were also not completely separated, although those sequences belonging to isolates of the clade SJP were sub-clustered inside the single branch of the tree (Figure 2d).

To obtain a broader perspective of phylogenetic relationship of the 5′ proximal region of the RNA2, *p15*, IR and the partial *MP* sequences were evaluated in a concatenated analysis. The generated tree showed a topology resembling that obtained using the IR (Figure 2e). Since *p15*, IR and *MP* occur on the same genomic component, the contrasted phylogenetic association between the mentioned isolates indicated recombination events along this RNA2 region.

### 3.4. Genetic Data Support the Occurrence of Two CiLV-C Clades

Genetic distances within and between the clades defined in the *p29* and *MP* trees were calculated using MEGA 6 [54]. Based on *p29* sequences, the genetic distance within isolates of the clade CRD (0.009 ± 0.006) and SJP (0.007 ± 0.004) were significantly lower than the values obtained comparing sequences from the two clades (0.184 ± 0.007) (*p* < 0.01). Similar trend was observed in the calculations using the *MP* gene *i.e.*, within the clade CRD (0.007 ± 0.006), within the clade SJP (0.004 ± 0.003) and between the clades (0.187 ± 0.006) (*p* < 0.01). Genetic distances based on the *p15* showed no significant differences whereas those from the IR sequences were not analyzed because of the high coefficient of variations observed in these data, particularly within members of the clade CRD.

Molecular variability of the studied CiLV-C population was also estimated calculating several population genetic parameters such as the nucleotide diversity (π) and the haplotype diversity (Hd) using DnaSP 5 [56]. Only sequences recovered from citrus hosts were considered in these assessments. Nucleotide diversity values ranged from 0.010 to 0.057, the higher values being those corresponding to *MP* (0.056 ± 0.009) and *p29* (0.053 ± 0.009), which were different (*p* < 0.05 and *p* < 0.1, respectively) to that showed by *p15* (0.010 ± 0.001) (Table 2). When the analysis was conducted using only the isolates clustered in the clade CRD, the π values associated to *MP* (0.007 ± 0.001) and *p29* (0.009 ± 0.001) were lower (*p* < 0.05 and *p* < 0.1, respectively) than those obtained including the whole population. In contrast, in the same analysis the values for *p15* (0.010 ± 0.001) and IR (0.016 ± 0.003) were kept invariable (*p* < 0.05). The haplotype diversity value for the IR reached maximum Hd = 1, indicating high variability of this region.

### 3.5. 5′-Proximal Region of CiLV-C RNA2 Harbors Recombination Signatures

To detect possible recombination events amongst the CiLV-C isolates, *p29*, *p15*, IR and *MP* sequences were analyzed using RDP4 v4.66 [60]. Sequences corresponding to the RNA2 (*i.e.*, *p15*-IR-*MP*) were concatenated before the analysis. Only those events consistently detected by four or more methods implemented in RDP4 and *p*-values < 0.05 were considered reliable results. Additionally, coding sequences (*p29*, *p15* and *MP*) were tested using GARD, implemented in the HyPhy package.

**Figure 3:**
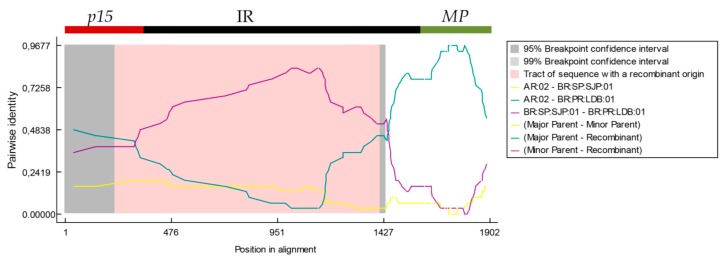
Evidence of recombination in RNA2 of the isolate BR_PR_LDB_01. Similarity plots of the recombinant with the minor (isolate BR_SP_SJR_01) and major (isolate AR_02) parents are shown as obtained from the analysis of concatenated sequences of the RNA2 (*p15* + IR + *MP*) using the RDP software.

No evidence of recombination was detected in *p29* and *MP* using both GARD and RDP4. However, in the analysis involving the RNA2 concatenated sequences, RDP4 revealed that the isolate BR_PR_LDB_01 (clade CRD) bears a putative recombination event supported by seven methods: RDP (*p* = 2.29 × 10^−4^), GENECONV (*p* = 1.29 × 10^−3^), BootScan (*p* = 3.75 × 10^−4^), MaxChi (*p* = 6.67 × 10^−7^), Chimaera (*p* = 4.19 × 10^−7^), SiScan (*p* = 1.55 × 10^−6^), and PhylPro (*p* = 2.1 × 10^−6^) (Figure 3). Recombinant sequence spanned from *p15* to almost the end of the IR and the isolates BR_SP_SJP_01 (clade SJP) and AR_2 (clade CRD) were indicated as probable minor and major parents, respectively. The ending breakpoint was identified in position 1180 in the concatenated sequence *p15*-IR-*MP,* which approximately corresponds to the position 1436 in the CiLV-C RNA2 complete sequence. The beginning breakpoint was not precisely determined by the software. Interestingly, evidence of the same recombinant events was detected in other 26 isolates of clade CRD indicating that all of them might be descendants from a common recombinant ancestor. It was noteworthy that the failure to determine the beginning breakpoints was constant in all detected recombinant events suggesting that identified parental isolates may be actually recombinants as well. Recombination between isolates of CiLV-C and CiLV-C2 was not detected.

### 3.6. Purifying Selection Is Acting on CiLV-C

The ratio between the non-synonymous and synonymous substitution frequencies (ω = dN/dS) in codons of the analyzed CiLV-C genes was estimated (Table 2). dN and dS were calculated using MEGA 6. In general, the ω values were lower than 1, indicating a negative or purifying selection, although a different level of pressure over each gene was observed. According to the results, *p29* (ω = 0.07) and *MP* (ω = 0.10) were exposed to stronger selection strengths than *p15* (ω = 0.50). Moreover, in the analysis when only the isolates of the clade CRD were included, ω value for *p15* kept almost invariable (ω = 0.57) whereas they were slightly augmented for *p29* (ω = 0.18) and *MP* (ω = 0.26).

Selection constraints operating on specific sites of the analyzed CiLV-C ORFs were estimated using SLAC (*p* < 0.1), FEL (*p* < 0.1) and REL (Bayes factor = 50) methods [59] implemented in HyPhy. Nucleotide substitution models TrN93 (Tamura-Nei) [68] was selected for the analyses of *p29*, HKY85 (Hasegawa, Kishino and Yano, 1985) [69] for *p15* and GTR (generalized time-reversible) for *MP*. General results reflected the ω values previously shown. In the sequence corresponding to the analyzed fragment of *MP*, 22 codons under purification strengths were identified using FEL and REL (Table 3), and the number of positions was reduced to three (codons 71, 112 and 148) when the SLAC result was integrated to the analysis. SLAC method tends to be more conservative among the three tools used, reducing the possibilities of identification of neutral sites as under selection [70]. In *p29*, ten codons were identified under negative selection by the three methods, and other 34 codons were also recognized as under purification using FEL and REL (Table 3). In *p15*, none of the positions was identified under negative selection, and only the codon at position 91 was indicated under positive selection using FEL and REL (Table 3).

### 3.7. Clade-Specific CiLV-C Isolates Can Be Differentially Detected by RT-PCR

**Figure 4:**
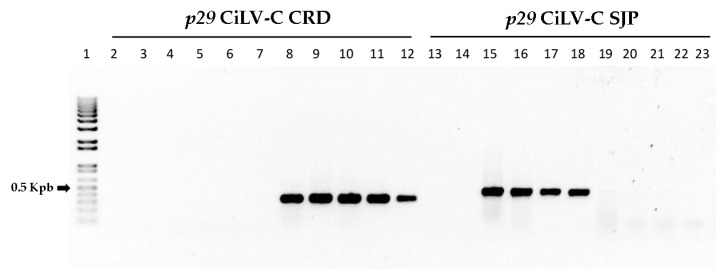
Differential detection of CiLV-C isolates belonging to clades CRD or SJP. 1% agarose gel electrophoresis of RT-PCR products obtained from nine CiLV-C isolates. *p29* CiLV-C CRD and *p29* CiLV-C SJP indicate reactions carried out using specific primers designed to detect isolates of the clade CRD and SJP, respectively. Lane 1: molecular weight marker, 1 Kbp Plus DNA Ladder (Invitrogen, Carlsbad, CA, USA); lanes 2 & 13: healthy citrus plant; lanes 3 & 14: citrus tree infected with CiLV-C2 isolate Colombia; lanes 4 & 15: Arabidopsis plant infected with CiLV-C isolate BR_SP_SJP_01; lanes 5 & 16: citrus plant from Cordeirópolis, SP (positive control sample for the clade CRD); lanes 6 & 17: citrus plant, São José do Rio Preto, SP (source of the isolate BR_SP_SJP_01—positive sample for the clade SJP); lanes 7 & 18: tropical spiderwort plant, São José do Rio Preto, SP (isolate BR_SP_SJP_04); lanes 8 & 19: citrus plant, Argentina (isolate AR_02); lanes 9 & 20: citrus plant, Londrina, PR (isolate BR_PR_LDB_01); lanes 10 & 21: citrus plant, Brasília, DF (isolate BR_DF_BSB_01); lanes 11 & 22: citrus plant, Maringá, PR (isolate BR_PR_MGF_01); lanes 12 & 23: citrus plant, Itu, SP (isolate BR_SP_ITU_01).

The viral isolates included in the present study were analyzed by RT-PCR using two primer pairs based on the *p29* gene. The test was designed to distinguish genotypes of the predefined clades CRD or SJP. As expected, amplicons of approx. 330 and 456 bp were observed from isolates previously identified as members of the clades CRD and SJP, respectively (Figure 4). Only one band per lane was observed indicating the absence of mixed infections. None of the primer pairs was able to detect CiLV-C2 from the positive control, which was amplified only with the specific primers previously reported [14] (Figure S1).

### 3.8. Genomic Characterization of CiLV-C Isolate BR_SP_SJP_01

**Figure 5:**
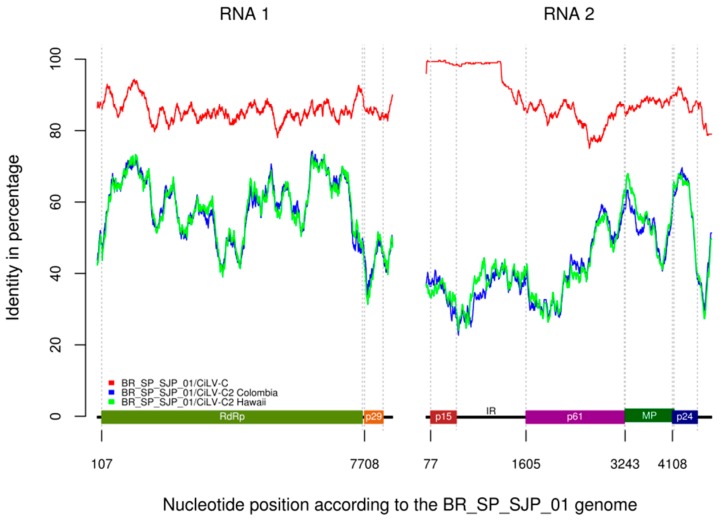
Nucleotide identity plot among BR_SP_SJP_01 full-length genome and those from CiLV-C, CiLV-C2 Colombia and CiLV-C2 Hawaii. Plots were generated with a sliding window size of 300 nt and a shift of one nucleotide at a time from aligned sequences. Nucleotide position is indicated at the beginning of each ORF according to the BR_SP_SJP_01 sequence.

To better understand the genetic characteristic of the viral haplotypes clustered in the clade SJP, the complete genome of one isolate representative of this clade was sequenced. RNA extracts were obtained from a sweet orange tree, the primary source of the isolate BR_SP_SJP_01, and from one Arabidopsis plant infected after the transmission experiments described below. Therefore, two sources of viral small RNAs were independently processed. The complete genome sequence of the isolate BR_SP_SJP_01 (GenBank database with accession numbers KP336746 and KP336747) was 13,757 nt in length, distributed in 8753 nt in RNA1 and 5004 nt in RNA2. The similarity profile generated from the RNA sequence alignments of CiLV-C and isolate BR_SP_SJP_01 revealed the presence of two peaks of maximum similarities in positions 500 and 1250 of the RNA1, approximately (Figure 5), which corresponded to the methyltransferase motif coding sequences conserved in a wide range of ssRNA viruses [71]. The profile of RNA2 showed a highly similar sequence spanning the first *ca.* 1400 nt of their 5′-proximal regions (Figure 5). In contrast, this region of BR_SP_SJP_01 showed the lowest identity (≈45%) with the tentative members of the genus *Cilevirus i.e.*, CiLV-C2 Co and CiLV-C2 Hw (Figure 5).

Globally, BR_SP_SJP_01 RNA1 and RNA2 showed 85.6% and 88.4% identity, respectively, with cognate molecules of CiLV-C (Table 4). Further comparisons revealed 85.4% nucleotide identity with the *RNA-dependent RNA polymerase* (*RdRp)* ORF and 85.0% with the *p29* ORF of this virus. A six nucleotide insertion was detected in the *p29* gene of the BR_SP_SJP_01 isolate, between positions 247–252 downstream of the translation start site. Nucleotide comparisons of the RNA2-encoded *p15*, *p61*, *MP* and *p24* ORFs showed 99.5%, 81.8%, 86.8% and 87.4% identity, respectively; whereas the IR had 96.7% identity with the equivalent sequences of CiLV-C. Although high values of nucleotide identity (>98%) were observed along the *p15* and IR regions, excluding these parts, the rest of the molecules showed 84.2% identity. In general, values of nucleotide identity between BR_SP_SJP_01 and those from CiLV-C2 were lower (44.7%–63.9%) than those observed in the comparison with CiLV-C (81.8%–99.5%) (Table 4).

The deduced amino acid sequence of RdRp from isolate BR_SP_SJP_01 had 93.1% identity with its equivalent from CiLV-C, whereas the predicted proteins from the ORFs *p24* and *MP* showed 93.9% and 91.9% identity, respectively. Proteins encoded by ORFs *p15* and *p61* showed the highest (100%) and the lowest (84.0%) values of identity, respectively. The putative coat protein P29 of the isolate BR_SP_SJP_01 (265 aa) was two amino acids longer than cognate protein encoded by CiLV-C (263 aa) and they shared 90.5% identity.

**Figure 6:**
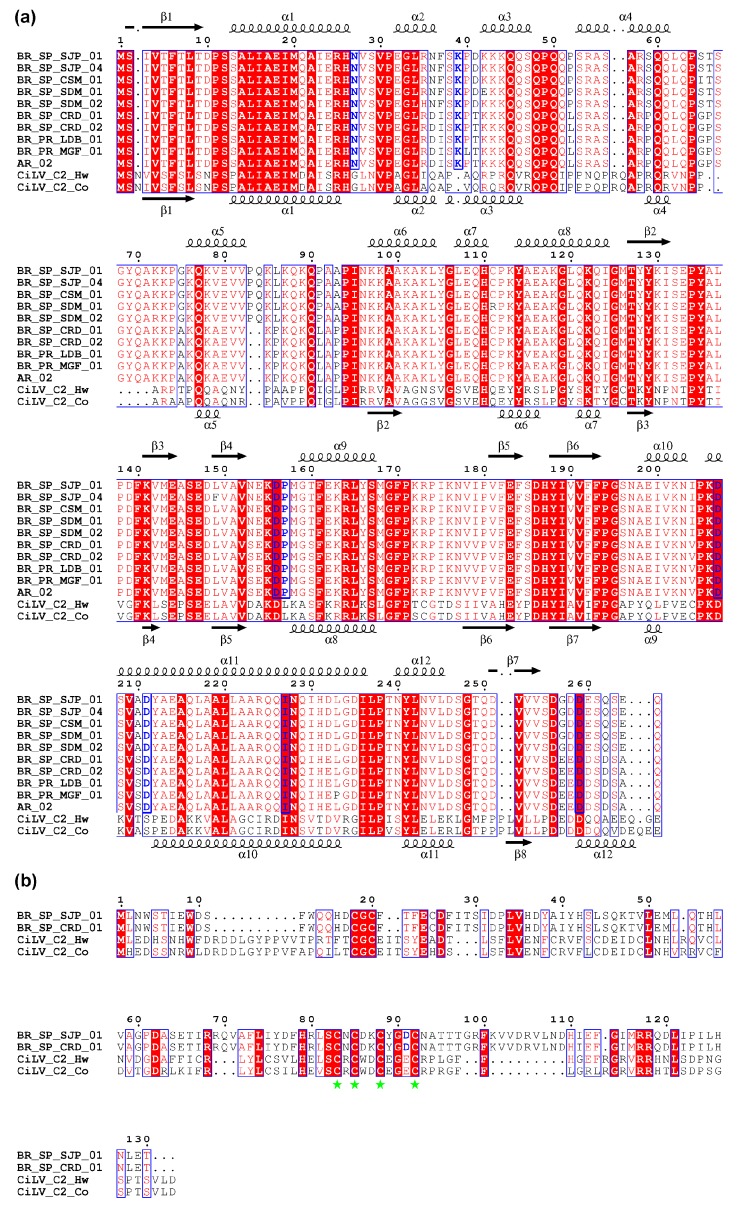
Deduced amino acid sequence alignments of P29 (**a**) and P15 (**b**) proteins from CiLV-C and CiLV-C2. Blue letters indicate positions under selection in CiLV-C as shown by a combination of SLAC, FEL and REL methods. (**a**) Consensus predicted secondary structures of P29 proteins from CiLV-C and CilV-C2 are on the top and bottom of each block, respectively. α, alpha-helix; β, beta strand; (**b**) Conserved Cys residues involved in a putative Zn-finger motif are highlighted with green stars.

To gain further understanding about the P29 protein of CiLV-C, deduced amino acids belonging to several isolates belonging to clades SJP and CRD were analyzed using the PROMALS algorithm, which generates sequence alignments using information from secondary structure prediction databases [61]. Similarly, available deduced amino acids sequences of the putative coat protein P29 from CiLV-C2 were analyzed using PROMALS. In an attempt to identify putatively conserved structural elements through the P29 proteins of tentative and definitive cileviruses, the results were compared to those obtained for CiLV-C. With the exception of a stretch between amino acids 50 to 120, predictions indicated a predominant array in alpha-helix connected by putative smaller beta stranded or non-ordinary secondary structure regions (Figure 6a). Pro83 and Gln84, introduced as consequence of six nucleotide insertion in *p29* genes of the clade SJP isolates were identified in the border of the putative alpha helix 5.

### 3.9. Mite-Mediated Transmission of the Isolate BR_SP_SJP_01

To verify the mite-mediated transmission of the isolate BR_SP_SJP_01, the assays were performed using as viral source the symptomatic sweet orange fruits from the tree where this isolate was originally obtained. After three days of acquisition, viruliferous mites were transferred to healthy Arabidopsis and sweet orange plants. Three independent experiments were conducted. Leprosis disease symptoms appeared 8–10 and 15–20 days after the inoculation in leaves of Arabidopsis and sweet orange plants, respectively. Transmission of CiLV-C isolate BR_SP_SJP_01 was confirmed in more than 90% of Arabidopsis and 93% of inoculated sweet oranges plants (Table S3).

## 4. Discussion

Citrus leprosis is considered a re-emergent disease in the Americas [1] and the most important among those with viral etiology affecting citrus orchards in Brazil. Since new viruses associated with the disease have recently been reported in Mexico and Colombia [12,13,14], the first objective of this work was to ascertain whether they occur in citrus trees showing typical leprosis symptoms in Brazil. Analysis of a discrete sample of symptomatic tissues collected from distant and diverse regions of the country during the period 2012–2015 indicated the absence of CiLV-C2 and CiLV-N. However, CiLV-C was detected in all of the evaluated samples confirming that this virus is the major causal agent for citrus leprosis in Brazil, as previously indicated [7,22].

We further studied the phylogeny and molecular diversity of CiLV-C as a way to assess the variability of the viral population and to elucidate putative forces driving its evolution. The sequences of four genomic regions (completed *p29* and *p15* genes, partial *MP* gene and the IR of the RNA2) of recently collected isolates were obtained and analyzed together with all of the CiLV-C sequences available in the GenBank database.

Phylogenetic analyses showed that based on *p29* and *MP* genes the CiLV-C population is consistently subdivided in two clades, where most of the Brazilian isolates are clustered along with the genotype of the type member virus of the genus *Cilevirus* [29], defining a group we called clade CRD. The other clade, called SJP, groups a few isolates identified in a small area of the central-northern part of the São Paulo State around the municipality of São José do Rio Preto.

The complete sequence of the isolate BR_SP_SJP_01, clade SJP, was obtained by next generation sequencing and its genomic organization is similar to that described for CiLV-C [32]. Overall, RNA1 of the isolate BR_SP_SJP_01 and CiLV-C shares 85.6% identity, while the value for the RNA2 segment is 88.4%. Higher identity value between the RNA2 of both isolates is due to the existence of a region of approx. 1400 nt in the 5′-termini of the molecules which show more than 98% nucleotide identity. Conversely, comparisons between isolate BR_SP_SJP_01 and the tentative member of the genus *Cilevirus i.e.*, CiLV-C2 isolates Co and Hw showed drastically reduced values of nucleotide identity. Differences were even greater in the 5′ proximal region of the RNA2 (ORF *p15* and IR), with values as low as 44.7%.

Recombination is considered one of the main sources for molecular variability in plant viruses [41,45]. Having acknowledged the widening disparities in the nucleotide identity along the RNA2 molecules of the isolate BR_SP_SJP_01 and the cilevirus type member, we used concatenated sequences derived from the RNA2 as a way to assess the putative RNA-RNA crossover among these isolates. Recombination was identified in the isolate BR_PR_LDB_01, and similar signatures were identified in other 26 isolates belonging to the clade CRD. Altogether, our findings suggest the *p15*-IR region as a crossover hotspot, indicating that recombination is an important source of genetic variability in natural populations of CiLV-C; and likewise, they raise a coherent framework to understand the observed phylogenetic relationship between the two clades in which the CiLV-C population is subdivided. It should be noticed, however, that existence of recombination in other regions of viral genome should not be excluded.

Recombination in RNA viruses seems to be promoted by transcriptional activity [72,73]. In *Brome mosaic virus* (BMV) an efficient recombination hotspot was mapped within the intercistronic region of RNA3, which drives the transcription of the sgRNA4 [72]. Similarly, the IR of cileviruses, which is upstream of ORF *p61*, could also harbor the promoter elements required to drive the expression of the *p61* subgenomic RNA transcribed during CiLV-C and CiLV-C2 replication [14,34].

High variation within the cilevirus RNA2 IR has been previously highlighted [14,21]. Overall, the region shows the lowest nucleotide sequence identity in pairwise comparisons. Moreover, the region harbors a putative ORF encoding a 7 kDa protein (P7) which is found 32, 160 and 515 nt downstream of *p15* gene in CiLV-C, CiLV-C2 Co and CiLV-C2 Hw, respectively; while it is partially duplicated in CiLV-C2 Hw [21]. In CiLV-C2, P7 shows a trans-membrane domain not observed in the protein encoded by CiLV-C. Although more evidences are required to understand the cileviruses evolution, it is possible to hypothesize that contrasting structural array among the IR of related viruses is a consequence of continuous illegitimate (non-homologous) recombination processes inter or intra species of cileviruses.

Nucleotide diversity of CiLV-C estimated from *p29* and *MP* (π*_p_*_29_ = 0.053 and π*_MP_* = 0.056) oscillated within the range observed for other citrus-infecting virus populations *i.e.*, *Citrus psorosis virus* (π = 0.083) [74], *Citrus tristeza virus* (π = 0.038) [75] and *Citrus leaf blotch virus* (π = 0.021) [76]. Nevertheless, molecular variability of CiLV-C population is mostly determined by the existence of isolates of the clade SJP. When sequences from these isolates were overlooked, π*_p_*_29_ and π*_MP_* values were reduced almost six-fold. Low variability seems to be constant along the CiLV-C genome, as also evidenced with the analyses of *p15*, and although to a lesser extent, with the IR sequences. Nucleotide diversities associated to *p15* and IR were similar when calculated for the whole population and for isolates of the clade CRD, a point likely reflecting the putative acquisition of this genomic region in an ancestral of these isolates from a member of the clade SJP by recombination. On the other hand, our results reveal that the subpopulation grouped in clade CRD, the prevalent through the extensive geographic area where CiLV-C occurs, is characterized by a low molecular variability. These results are reinforced by the fact that the study involved citrus-infecting isolates collected in remote areas of Brazil, and in the case of the analysis of the *MP*, also incorporated isolates from Mexico to Argentina. Consistently with this, the analysis of the recently obtained sequence of a CiLV-C isolate originally collected in Argentina in 1967 [77] also supports our findings. This isolate was recovered from the peel of orange fruits showing leprosis-like symptoms that were conserved in the herbarium of the United States Department of Agriculture (USDA) inspection stations [77]. Remarkably, after recovering 87.0% and 98.0% of the RNA1 and RNA2 viral genomic molecules, respectively, the sequences of the CiLV-C isolate Argentina 1967 showed more than 99% nucleotide identity with their cognates from the type member of the genus *Cilevirus* [77]. This suggests not only low spatial, but also low temporal variability within CiLV-C isolates.

Low genetic variability seems to be common in natural plant virus populations, which is probably associated with continuous genetic bottlenecks *i.e.*, infection of a new cell or vector transmission [39,45,46]. In this regard, it seems plausible that some traits of the CiLV-C biology contribute to low genetic variability and potentiate the impact of bottlenecks on the viral population diversity. Even though plant species belonging to 28 families can be experimentally infected by CiLV-C, the known natural host range of the virus is limited to a few species (tropical spiderwort, citrus and *Swinglea glutinosa*) and in both natural and experimental hosts, the virus does not spread systemically [17,26,27,78,79]. Consequently, after multiplication in a relatively low number of epidermal and parenchymal cells around the mite feeding sites [8,26,27,80], CiLV-C colonization to distal parts of the infected plant is exclusively mediated by viruliferous mites. Recently, new evidence indicating multiplication of cileviruses in mites have been suggested [1]. In that context, alternate replication in plant and mite cells might further constrain virus variability as previously observed in the arthropod-borne viruses *Rice dwarf virus* (RDV) [81] and *Tomato spotted wilt virus* (TSWV) [82].

Low diversity of natural host range of CiLV-C, noticeably homogenous in the context of large-scale sweet orange cultivation, and mite-mediated transmission may also account for the negative or purifying selection mechanism operating upon the evolution of CiLV-C. dN/dS values calculated for p29, MP and p15 were lower than 1, but lower values corresponding to p29 and MP suggested the proteins encoded by these two genes are less flexible to putative amino acids changes than the protein P15 (Table 2). Purifying selection is the main evolutionary force acting on numerous plant viruses as result of structural constraints and preservation of specific molecular interactions [45]. Particularly, P29 and MP of CiLV-C seem to follow the rule since they may at least be involved in virus particle formation, virus-vector interaction and cell-to-cell movement. Role of P15 in the viral multiplication cycle remains unknown, therefore we lack information to discuss the selection data obtained. However, it is worth noting that several Cys residues are highly conserved among P15 from CiLV-C and CiLV-C2 and they might be involved in a putative Zn-finger motif (Figure 6b). Remarkably, the site encoding the Asp91 in p15 gene from CiLV-C and identified under selection (Table 3) is placed inside the loop of the predicted Zn-finger motif where it aligns with a similar negatively charged amino acid (Glu) in CiLV-C2.

In accordance with dN/dS values for *p29* and *MP* genes, several negatively selected sites were identified in these regions (Table 3). In *MP*, a total of 22 sites were detected as negatively selected and several of them are involved in conserved sequences and structural motifs of plant virus movement proteins of the 30K superfamily according to a recent study [83] (e.g., Ile84 and Ser85 in the beta strand 2; Val92, Pro93, Pro98 and Ala99 in the loop 2; Ser102 and Lue103 in the beta strand 3; Leu112 in the loop 3; and Val115 and Gln121 in the strand 4). In *p29*, many negatively selected sites were revealed to be conserved through the homologous proteins from CiLV-C and CiLV-C2. Analysis revealed that seven out of the ten positions recognized under negative selection by using SLAC, REL and FEL methods are distributed in the C-terminal half of the proteins and five of them encode charged amino acids (Asp156, 207, 211, 259 and Arg173). Four out of the ten amino acids are part of predicted alpha-helix structures throughout the four homologous proteins (Figure 6a). Coat proteins from plus strand RNA viruses are involved in several functions during replication, vector interaction and symptom development, determining the viral pathogenicity and epidemiology [84]. In this regard, our studies suggest that amino acids encoded by selected codons may play important functional or structural roles and delimit the sites that can be potentially mutated to assay the properties of proteins and the course of virus infection. Further analyses with mutant infectious clone of CiLV-C will allow final determination of the significance of amino acids under selection pressure.

CiLV-C isolate BR_SP_SJP_01 was transmitted from symptomatic sweet orange fruits to sweet orange seedlings and Arabidopsis plants by viruliferous *B. yothersi* mites. The high number of symptomatic plants observed and the confirmation of viral presence at molecular level indicated that mite mediated transmission process of this isolate is at least as efficient as previously described for isolates of the CiLV-C type member [85]. Analyses of symptoms, vector transmission and plant host range did not reveal contrasting biological characteristics between the isolate BR_SP_SJP_01 and the CiLV-C type member. Currently, nucleotide identity-based threshold for species demarcation in the genus *Cilevirus* has not been implemented and biological criteria for distinguishing among these viruses have not been recognized. However, based on genome identity data shown elsewhere [14], it seems to be obvious that at least two different viral species exist associated with citrus leprosis within the genus *Cilevirus i.e.*, CiLV-C and CiLV-C2 (55% nucleotide identity between their genomes). By analysing this in greater depth, CiLV-C2 isolate Colombia shows about 85% nucleotide identity with CiLV-C2 isolate Hawaii, and they may represent two clades of the same species separately evolving in distant geographic regions and, at least to date, in different hosts (isolate Colombia in sweet orange and isolate Hawaii in hibiscus). CiLV-C isolate BR_SP_SJP_01 (clade SJP) and CiLV-C type member, epitomized by the isolate BR_SP_CRD_01 (clade CRD), also show 85% to 88% nucleotide identity among their genomes, but differently from that observed in the case of CiLV-C2, the two isolates of CiLV-C coexist in the same geographic area and share the same natural hosts. Probably, forces beyond genetic drift, such as those intrinsic to the vector transmitting the virus, might account for the origin of these two divergent genotypes within the CiLV-C population. Interestingly, recent studies showed that several mite morphotypes previously identified as members of the *B. phoenicis* species complex are indeed new species [25] and, on top of that, a new report disclosed the existence of diverse *Brevipalpus* populations in Brazil [86].

Finally, the low molecular variability observed within the *MP* gene in this study warrants the use of the traditional detection test of CiLV-C based on its partial amplification [51]. In addition, we described two primer pairs based on the *p29* gene sequence which differentially detect the presence of CiLV-C isolates belonging to the two phylogenetic clades identified in this work. Information and tools generated in this work in combination with those arising from *Brevipalpus* population studies will probably lead to improving the strategies used for the control of the citrus leprosis disease, while residues putatively relevant for the biological function of viral proteins were also revealed.

## 5. Conclusions

This study describes the *Citrus leprosis virus C* population as having overall low genetic variability although divided in two clades named CRD and SJP. The complete nucleotide sequence of a new isolate from the clade SJP, which naturally infects not only citrus but also the common weed *Commelina benghalensis,* was obtained. This isolate is efficiently transmitted by *Brevipalpus yothersi* Baker (synonym *B. phoenicis* Geijskes citrus type). At the protein level, several amino acids putatively relevant for cileviruses life cycle were found to be affected by negative selection.

## Figures and Tables

**Table 1:** Complete list of CiLV-C sequences used in this work. Accession numbers in the GenBank database for genomic region, host and year of collection of each viral isolates are indicated.

Isolate Identification ^1^ and Host ^2^ of Collection	Collection Year	Viral Genomic Region	GenBank Accession No.	Reference
Brazilian isolates				
BR_MG_AFE_01	2012	*p29*	KR093040	This work
BR_SP_AMP_01	2012	*p29*	KR093041	This work
		*p15*	KR093078	
		IR	KR093139	
		*MP*	KR093106	
BR_SE_AJU_01	2012	*p29*	KR093042	This work
		*p15*	KR093079	
		IR	KR093140	
		*MP*	KR093107	
BR_SP_ARA_01	2014	*p29*	KR093043	This work
		*p15*	KR093080	
		IR	KR093141	
		*MP*	KR093108	
BR_PA_BEL_01	2012	*p29*	KR093045	This work
		*p15*	KR136415	
		IR	KR093143	
		*MP*	KR093110	
BR_SP_BRM_01 (*Commelina benghalensis*)	2012	*MP*	JQ944802	[49]
BR_SP_BRM_02	2012	*p29*	KR093046	This work
		*p15*	KR093084	
		IR	KR093144	
		*MP*	KR093112	
BR_DF_BSB_01	2012	*p29*	KR093047	This work
		*p15*	KR093085	
		IR	KR093145	
		*MP*	KR093113	
BR_SP_CMP_01	2012	*p29*	KR093049	This work
BR_SP_CSB_01	2012	*p29*	KR093050	This work
		*p15*	KR093086	
BR_SP_CLN_01	2012	*p29*	KR093051	This work
		*p15*	KR093082	
BR_MG_CGZ_01	2012	*p29*	KR093048	This work
		*p15*	KR093087	
		IR	KR093146	
		*MP*	KR093114	
BR_SP_CCH_01	2012	*p29*	KR093052	This work
		*p15*	KR093088	
		IR	KR093147	
		*MP*	KR093115	
BR_SP_CRD_01	2006	RNA1	DQ352194	[32]
		RNA2	DQ352195	
BR_SP_CRD_02	2012	*p29*	KR093053	This work
		*p15*	KR093089	
		IR	KR093148	
		*MP*	KR093116	
BR_SP_CRD_03	2012	*MP*	KR093117	This work
BR_SP_CSM_01	2012	*p29*	KR093054	This work
		*p15*	KR093083	
		IR	KR093149	
		*MP*	KR093111	
BR_GO_GYN_01	2012	*p29*	KR093055	This work
		*p15*	KR093090	
		IR	KR093150	
		*MP*	KR093118	
BR_MG_LAV_01	2012	*MP*	KR093120	This work
BR_SC_LSP_01	2012	*p29*	KR093056	This work
		*p15*	KR093091	
		IR	KR093151	
		*MP*	KR093119	
BR_PR_LDB_01	2012	*p29*	KR093057	This work
		*p15*	KR093092	
		IR	KR093152	
		*MP*	KR093121	
BR_AM_MAO_01	2012	*p29*	KR093058	This work
		*p15*	KR093093	
		IR	KR093153	
		*MP*	KR093122	
BR_PR_MGF_01	2012	*p29*	KR093059	This work
		*p15*	KR093094	
		IR	KR093154	
		*MP*	KR093123	
BR_SP_MRN_01	2012	*p29*	KR093060	This work
		*p15*	KR136416	
		IR	KR093155	
		*MP*	KR093124	
BR_SC_NCH_01	2012	*p29*	KR093061	This work
BR_SC_NTB_01	2012	*p29*	KR093062	This work
BR_TO_PMW_01	2012	*p29*	KR093063	This work
		*p15*	KR093105	
		IR	KR093166	
		*MP*	KR093134	
BR_SP_PRB_01	2014	*p29*	KR093064	This work
		*p15*	KR093095	
		IR	KR093156	
		*MP*	KR093125	
BR_GO_PNT_01	2012	*p29*	KR093065	This work
		*p15*	KR093096	
		*MP*	KR093126	
BR_SP_PRT_01	2012	*p29*	KR093066	This work
		*p15*	KR093097	
		IR	KR093157	
		*MP*	KR093127	
BR_AC_RBR_01	2012	*p29*	KR093067	This work
BR_SP_ITU_01	2012	*p29*	KR093070	This work
		*p15*	KR093099	
		IR	KR093159	
		*MP*	KR093129	
BR_MG_STC_01	2012	*p29*	KR093069	This work
BR_SP_SAP_01	2012	*p29*	KR093068	This work
		*p15*	KR093098	
		IR	KR093158	
		*MP*	KR093128	
BR_SP_JBT_01	2006	RNA1	DQ157466	[34]
		RNA2	DQ157465	
BR_SP_SNG_01	2012	*p29*	KR093071	This work
		*p15*	KR093100	
		IR	KR093160	
		*MP*	KR093130	
BR_SP_SJP_01	2012	RNA1	KP336746	This work
		RNA2	KP336747	
BR_SP_SJP_02	2012	*p29*	KR093072	This work
		*p15*	KR093101	
		IR	KR093161	
		*MP*	KR093135	
BR_SP_SJP_03	2012	*p15*	KR093167	This work
		IR	KR093162	
		*MP*	KR093136	
BR_SP_SJP_04 (*Commelina benghalensis*)	2013	*p29*	KR093077	This work
		*MP*	KR093137	
BR_SP_SDM_01	2015	*p29*	KT253463	This work
		*p15*	KT253469	
		IR	KT253475	
		*MP*	KT253481	
BR_SP_SDM_02	2015	*p29*	KT253464	This work
		*p15*	KT253470	
		IR	KT253476	
		*MP*	KT253482	
BR_SP_SDM_03	2015	*p29*	KT253465	This work
		*p15*	KT253471	
		IR	KT253477	
		*MP*	KT253483	
BR_SP_SDM_04	2015	*p29*	KT253466	This work
		*p15*	KT253472	
		IR	KT253478	
		*MP*	KT253484	
BR_SP_SDM_05	2015	*p29*	KT253467	This work
		*p15*	KT253473	
		IR	KT253479	
		*MP*	KT253485	
BR_SP_SDM_06	2015	*p29*	KT253468	This work
		*p15*	KT253474	
		IR	KT253480	
		*MP*	KT253486	
BR_RJ_TNG_01	2012	*p29*	KR093073	This work
		*p15*	KR093102	
		IR	KR093163	
		*MP*	KR093131	
BR_SP_TTI_01	2012	*p29*	KR093074	This work
		*p15*	KR093103	
		IR	KR093164	
		*MP*	KR093132	
BR_MT_TRN_01	2012	*p29*	KR093075	This work
		*p15*	KR093104	
		IR	KR093165	
		*MP*	KR093133	
BR_MG_UBA_01	2012	*p29*	KR093076	This work
		*MP*	KR093138	
Isolates from other countries				
AR_01	2012	*MP*	JX163907	[8]
AR_02	2012	*p29*	KR093044	This work
		*p15*	KR093081	
		IR	KR093142	
		*MP*	KR093109	
CO_01	2005	*MP*	DQ272491	[10]
MX_01	2010	*MP*	HQ292778	[9]
PA_01	2006	RNA1	DQ388512	[50]
		RNA2	DQ388513	
PY_01	2012	*MP*	JX163908	[8]

^1^ Unless otherwise indicated, the collection hosts for isolates described in this work were sweet orange (*Citrus sinensis* L. Osb.) trees; ^2^ Isolates were designated including several entries separated by underscores. First two letters identify the country (AR: Argentina; BR: Brazil; CO: Colombia; MX: Mexico; PA: Panama and PY: Paraguay), followed by the Brazilian state (AC: Acre; AM: Amazonas; DF: Distrito Federal; GO: Goiás; MG: Minas Gerais; MS: Mato Grosso do Sul; PA: Pará; PR: Paraná; RJ: Rio de Janeiro; SC: Santa Catarina; SE: Sergipe; SP: São Paulo and TO: Tocantins), the city of collection (AFE: Alfenas; AJU: Aracaju; AMP: Amparo; ARA: Araras; BEL: Belém; BRM: Borborema; BSB: Brasília; CCH: Conchal; CRD: Cordeirópolis; CGZ: Comendador Gomes; CLN: Colina; CMP: Campinas; CSB: Casa Branca; CSM: Cosmorama; GYN: Goiânia; ITU: Itu; JBT: Jaboticabal; LAV: Lavras; LDB: Londrina; LSP: Linha Espuma; MAO: Manaus; MGF: Maringá; MRN: Mirandópolis; NCH: Nova Erechim; NTB: Nova Itaberaba; PMW: Palmas; PNT: Planaltina; PRB: Piracicaba; PRT: Pratânia; SAP: Santo Antônio de Posse; SDM: Sud Mennucci; SJP: São José do Rio Preto; SNG: Serra Negra; STC: Santa Conceição; TNG: Tanguá; TRN: Terenos; TTI: Tatuí; UBA: Uberaba; UBT: Ubatuba), and the sample number.

**Table 2:** Population genetic parameters and selection pressure estimates for four CiLV-C genomic regions. Independent analyses were carried out including all isolates and only those grouped within the clade CRD according to the phylogenetic study.

Genomic Region	Region Length (nt)	Dataset ^1^/# of Isolates	π	h	Hd	dS	dN	ω (dN/dS)
***p29***	792	All/47	0.053 ± 0.009	40	0.990	0.252 ± 0.037 ^2^	0.018 ± 0.003 ^2^	0.07
		CRD/38	0.009 ± 0.001	32	0.986	0.022 ± 0.004	0.004 ± 0.001	0.18
***p15***	393	All/41	0.010 ± 0.001	30	0.961	0.014 ± 0.004	0.007 ± 0.002	0.50
		CRD/31	0.010 ± 0.001	24	0.953	0.014 ± 0.004	0.008 ± 0.002	0.57
***MP***	300	All/46	0.056 ± 0.009	22	0.854	0.202 ± 0.043	0.021 ± 0.007	0.10
		CRD/36	0.007 ± 0.001	17	0.778	0.019 ± 0.006	0.005 ± 0.002	0.26
**IR**	940	All/38	0.021 ± 0.003	38	1	-	-	-
		CRD/28	0.016 ± 0.003	28	1	-	-	-

^1^ Only citrus isolates were considered. ^2^ Standard error of means was calculated by using the bootstrap method implemented in the MEGA6 program. All: Total population; CRD: Only members of the clade CRD; π: Nucleotide diversity; h: Number of haplotypes; Hd: haplotype (gene) diversity; dS: frequency of synonymous substitution per site; dN: frequency of non-synonymous substitution per site.

**Table 3:** Amino acids likely to be under selection in CiLV-C.

ORF	Nucleotide Substitution Model	Methods	Selection Pressure	Amino Acid Position in the Sequence of Isolate BR_SP_SJP_01
***p29***	TrN	SLAC-FEL-REL	purification	27, 39, 113, 156, 157, 173, 207, 211, 227, 259
		FEL-REL	purification	2, 7, 23, 30, 47, 49, 57, 58, 76, 87, 99, 100, 104, 116, 118, 133, 137, 140, 151, 154, 165, 171, 172, 180, 181, 190, 196, 202, 213, 214, 217, 219, 245, 254
***p15***	HKY85	SLAC-FEL-REL	-	-
		FEL-REL	positive	91
***MP*^1^**	GTR	SLAC-FEL-REL	purification	71, 112, 148
		FEL-REL	purification	59, 74, 80, 84, 85, 92, 93, 96, 97, 98, 99, 102, 103, 111, 115, 118, 121, 136, 146

^1^ Fragment of the gene corresponding to amino acid stretch between the positions 55 and 150.

**Table 4:** Nucleotide and deduced amino acids identities between the isolate BR_SP_SJP_01 and definitive and tentative cileviruses.

BR_SP_SJP_01	CiLV-C		CiLV-C2 Isolate Colombia		CiLV-C2 Isolate Hawaii	
	nt	aa	nt	aa	nt	aa
**RNA1**	85.6	-	60.1	-	60.1	-
ORF *RdRp*	85.4	93.1	61.4	59.3	61.4	59.1
ORF *p29*	85.0	90.5	49.2	36.0	49.0	35.0
**RNA2**	88.4	-	52.6	-	52.0	-
ORF *p15*	99.5	100.0	44.7	20.8	45.2	24.6
ORF *p61*	81.8	84.0	52.0	32.0	51.2	33.1
ORF *MP*	86.8	91.9	56.6	51.7	54.9	51.4
ORF *p24*	87.4	93.9	63.9	63.1	62.0	61.6
IR	96.7	-	46.0	-	46.0	-

## Supplementary Materials

The following are available online at www.mdpi.com/1999-4915/8/6/153/s1, Figure S1: 1% agarose gel electrophoresis of RT-PCR products for detection of leprosis associated viruses; Table S1: Library composition and viral genome coverage of the isolate BR_SP_SJP_01 obtained by next generation sequencing of the small RNA fractions derived from infected sweet orange and Arabidopsis plants; Table S2: List of primers used to amplify the BR_SP_SJP_01 complete genome; Table S3: Transmission efficiency of CiLV-C isolate BR_SP_SJP_01 by *Brevipalpus yothersi* mites.
