# Supplementary material for: Phylogenetic and Molecular Variability Studies Reveal a New Genetic Clade of Citrus leprosis virus C
Source: Viruses. 2016 Jun 6;8(6):153. doi: 10.3390/v8060153 (PMC4926173; doi:10.3390/v8060153)
Supplement: Supplementary file 1 [file viruses-08-00153-s001.pdf]

Supplementary Materials: Phylogenetic and Molecular Variability Studies Reveal a New Genetic Clade of *Citrus leprosis virus C*

Pedro Luis Ramos-González, Camila Chabi-Jesus, Orlene Guerra-Peraza, Michèle Claire Breton, Gabriella Dias Arena, Maria Andreia Nunes, Elliot Watanabe Kitajima, Marcos Antonio Machado and Juliana Freitas-Astúa

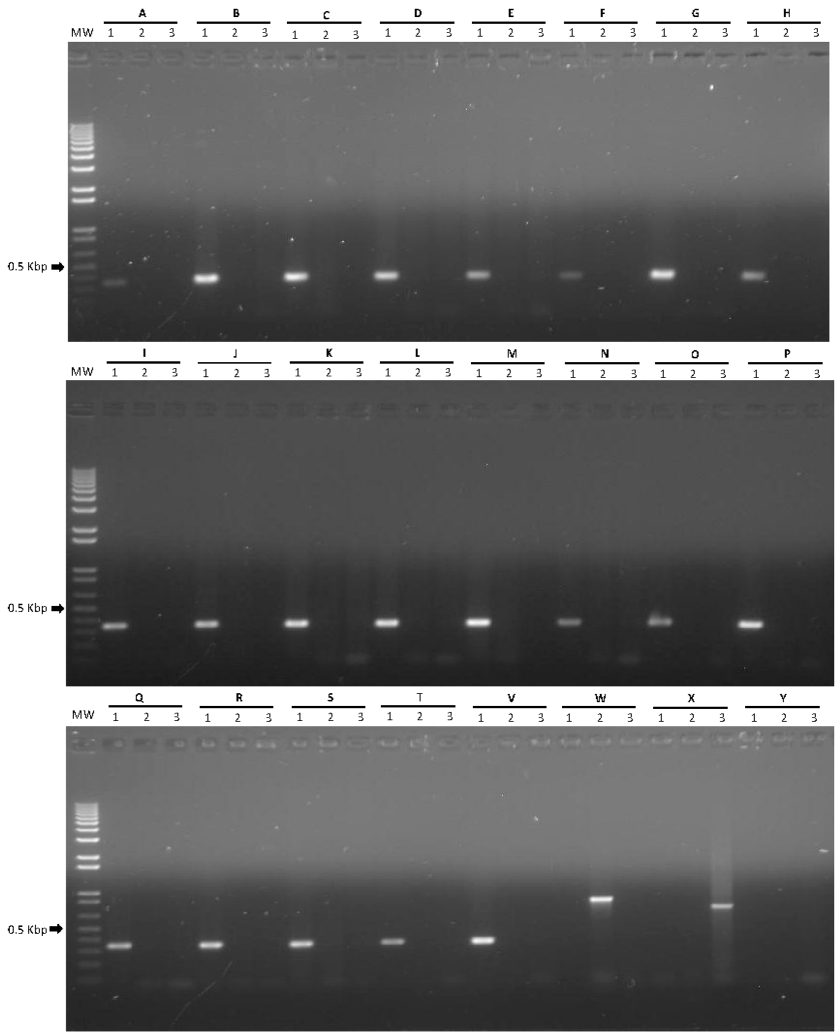

Figure S1. 1% agarose gel electrophoresis of RT-PCR products for detection of leprosis associated viruses. Each RNA sample was analyzed with three independent primer pairs which are specific for the detection of CiLV-C (partial *MP* gene, lanes 1), CiLV-C2 (*p29* gene, lanes 2), and CiLV-N (partial *N* gene, lanes 3). Each isolate was identified with a letter. A: BR_PR_LDB_01, B: BR_GO_PNT_01, C: BR_AM_MAO_01, D: BR_GO_GYN_01, E: BR_MG_CGZ_01, F: BR_MT_TRN_01, G: BR_DF_BSB_01, H: BR_TO_PMW_01, I: BR_RJ_TNG_01, J: BR_PA_BEL_01, K: BR_SE_AJU_01, L: BR_SC_LSP_01, M: BR_PR_MGF_01, N: BR_SP_PRB_01, O: BR_SP_ARA_01, P: BR_SP_MRN_01, Q: BR_SP_SJP_01, R: BR_SP_CSB_01, S: BR_SP_CRD_02, T: BR_SP_CSM_01, V: CiLV-C – infected sweet orange plant (positive control), W: CiLV-C2– infected citrus from Meta, Colombia (positive control), X: CiLV-N infected citrus from Guadalajara, México (positive control), Y: Healthy citrus plant. MW: molecular weight marker, 1 Kbp Plus DNA Ladder (Invitrogen, Carlsbad, CA, USA). Sizes of the expected amplicons are 339, 795 and 655 bps, for CiLV-C, CiLV-C2, and CiLV-N, respectively. Figure shows 20 of the 46 isolates analyzed in this work (Table 1).

Table S1. Library composition and viral genome coverage of the isolate BR_SP_SJP_01 obtained by next generation sequencing of the small RNA fractions derived from infected sweet orange and Arabidopsis plants.

	***Citrus sinensis* library**		***Arabidopsis thaliana* library**	
Total of reads (20–25 nt in length)	4,693,816		2,264,321	
	Isolate BR_SP_SJP_01 [1]			
	RNA1	RNA2	RNA1	RNA2
Viral RNA matched reads	1,829,215	579,817	868,813	812,968
Non-specific RNA matched reads	2,864,601	4,113,999	1,395,508	1,451,353
Total of bases in viral RNA matched reads	40,291,858	12,775,407	19,878,115	18,582,061
Viral RNA length (nt)	8753	5004	8753	5004
Viral RNA covered (%)	98.0	86.0	94.0	98.0
Viral genome coverage	4603.9	2552.6	2271.0	3713.9

[1] Isolate BR_SP_SJP_01 sequences are deposited in the GenBank under the accession numbers KP336746 and KP336747.

Table S2. List of primers used to amplify the BR_SP_SJP_01 complete genome.

Primer Sequence (5′–3′)	Direction [1]	Target Region	Amplicon Size (bp)
**RNA1**			
GTCAAGTGATATACCATTTTGCTTG	F	9–33	666
TCATCGTCTTTTCTGTAACCG	R	655–665	
CGCACATAATAACGCTGCTG	F	595–614	677
GCAACAGAACCAAACATCTC	R	1259–1278	
TGTTCATAGGTTGGATGTGT	F	1171–1191	652
AGCAGATGATGGATGTCGG	R	1804–1820	
CGGTATAGTTCGGTTCCTTA	F	1750–1768	789
ACAAGCAAGCTGTAATGACT	R	2489–2508	
CCCATTTCATCTCAAATAAAGGC	F	2436–2458	685
CTAAATACCGCAGTTCCACC	R	3101–3121	
TTCACTTGGTTTTAGGACGC	F	3022–3041	670
ACGTTCATGTGTAGCAGAAG	R	3672–3691	
ACTTGAATCCGAATGCTTACA	F	3595–3615	688
AAAGATTACTGCCGGCTTTT	R	4263–4282	
TAAGGCTATCGTTGGTGTTG	F	4201–4221	670
GCGGTTCTCAACAGTTGG	R	4853–4871	
CCTTGTTGGTCGATACTTTCT	F	4809–4829	664
AAGATTGAACCACCACTACTT	R	5452–5472	
GAAAGATAGATTTGACCATGACG	F	5377–5399	665
TTAGCTGCATGACCTCAAAC	R	6022–6041	
TGCTTAAATCTATTGTCGGTCA	F	5952–5974	660
CCCCAATCACCTCATCAAC	R	6592–6611	
CGCAATGTTCCCCAATTATC	F	6434–6453	767
TCATCACCGGAAAATACTGC	R	7181–7200	
ACACTGTTCCTTATGGCTTT	F	7124–7143	651
TAGCCTGCATAATCTCAGCA	R	7755–7774	
ACCGTGAATTTGTATTTTGTCA	F	7686–7706	657
GTCAGCAACGGAATCCTTAG	R	8321–8341	
ACTGCCCTAAGTATGCTGAA	F	8036–8055	653

CAGCTGGAAGAGACTAGAAA	R	8668–8687	
**RNA2**			
TGTTCTAGGCTAATAACTCTCAA	F	36–58	
ATAAATCTAATCAAACTATTGTATTGTTCTAGGC [2]	F	11–45	667–692
CTGAAACAGCTCATGAAACA	R	684–703	
ACTTGTGTTTGTCATTTGCC	F	608–627	464
ATCGGCTTTATGACCTTACC	R	1052–1071	
TATGTATTGAAGCGCGGTTT	F	931–951	657
GCTTGATTTGTTGTAGGCTC	R	1568–1586	
CAACCGGTTTGTACATATTGT	F	1492–1512	576
AAGCCTTTGGTATTGAGTGA	R	2048–2067	
GGGCAAGTACATATTGGTGA	F	1971–1991	682
AACAACCAGATGATGTACAGA	R	2632–2652	
TTCTGTGTCAATTATTCTATGCA	F	2562–2584	650
TCAATGGTATCAGCTATGTCG	R	3191–3211	
GTTACTCCCAACTTACACTCA	F	3089–3109	667
AACACGACCCGAAATGTTAT	R	3736–3755	
GACAGGACTCATGTAAACGG	F	3669–3688	550
CTGCTTGATGTCGGCAAC	R	4201–4218	
CGAATAAACGCCTTTTGAGG	F	4130–4149	813
CCACCAGCTAGAAGAGACTA	R	4921–4943	

[1] F: forward and R: reverse; [2] Two partially overlapped forward primers were used to amplify this fragment.

Table S3. Transmission efficiency of CiLV-C isolate BR_SP_SJP_01 by *Brevipalpus yothersi* mites.

Host	Experiment	Number of Infected/Inoculated Plants [1]	Percentage of Infected Plants
Arabidopsis thaliana	1	15/15	100
	2	15/15	100
	3	14/15	93.3
Citrus sinensis cv. Pera	1	10/10	100
	2	9/10	90
	3	10/10	100

[1] Virus presence was confirmed by RT-PCR using primers that detect part of the CiLV-C *MP* gene.

© 2016 by the authors; licensee MDPI, Basel, Switzerland. This article is an open access article distributed under the terms and conditions of the Creative Commons by Attribution (CC-BY) license (http://creativecommons.org/licenses/by/4.0/).
